# Supplementary material for: StHsfB5 Promotes Heat Resistance by Directly Regulating the Expression of Hsp Genes in Potato
Source: Int J Mol Sci. 2023 Nov 20;24(22):16528. doi: 10.3390/ijms242216528 (PMC10671264; doi:10.3390/ijms242216528)
Supplement: Supplementary file 1 [file ijms-24-16528-s001.zip › ijms-2642863-supplementary.pdf]

Table S1. Primers used in the manuscript.

Primer name	Sequences	purpose
StHsfB5-OE-F	5'GGACTAGTATGGCCACAGATAATCGGAAT3'	Constructing overexpression plasmid HsfB5-pCAMBIA1305
StHsfB5-OE-R	5'CGGGATCCAAACAACCTTCTAGTTTTATTATGT3'	
StHsfB5-SRDX-F	5'GGACTAGTATGGCCACAGATAATCGGAAT3'	Constructing overexpression plasmid HsfB5-SRDX-pCAMBIA1305
StHsfB5-SRDX-R	5'CGGGATCCagcgaaacccaaacggagttctagatcgagatctaa AAACAACCTTCTAGTTTTATTATGT3'	
StHsfB5-IF	5'ATGACGCACAATCCCACTAT3'	PCR identification for StHsfB5-pCAMBIA1305 lines
StHsfB5-IR	5'TAGGAAATACACTTGGCTCACA3'	
StHsfB5-qRT-F	5'GAAAAGTTCCAGAAAGGGTGC3'	qRT-PCR
StHsfB5-qRT-R	5'CCATGAGAAGTTGCCTTGTTGTA3'	
EF1α-F	5'CTGTTAAGGATCTGAAGCGTGGT3'	qRT-PCR
EF1α-R	5'AATGTGGGAAGTGTGGCAGTCG3'	
sHsp17.6-F	5' AGCGGAGAGAGGAATGTGGAGAAAG3'	qRT-PCR
sHsp17.6-R	5'CAGGCTTCTTCACTTGTTCCTTTGG3'	
sHsp21-F	5'CTTGCTTTTAATGGAGGTTGGGTTG3'	qRT-PCR
sHsp21-R	5' TCCTCTTTTCCATTATTCTTGACCC3'	
sHsp22.7-F	5' TGGCCTTATTAGCTCGTTCAGATTG3'	qRT-PCR
sHsp22.7-R	5'CTCTCAACTCTATGCCATTTCTCACC3'	
Hsp80-F	5'TCGTCGTTTCAGACCGTGTTGTG3'	qRT-PCR
Hsp80-R	5'GTCATTCTTGTCTGCATCAGCCCTC3'	
StHsfB5-ADF	5'GGCCATGGAGGCCATGGCCACAGATAATCGGAAT3'	Constructing HsfB5-pGADT7
StHsfB5-ADR	5'CCCCCGGG CTAAAACAACCTTCTAGT3'	
sHsp21-HSE-F	5'AGCTTCCTTCTACCAATATCCTAGAACTTTCTGGGTAC3'	Constructing pro-sHsp21-pABAi
sHsp21-HSE-R	5'C CAGAAAGTTCTAGGATATTGGTAGAAGGA3'	

sHsp22.7-HSE-F	5'AGCTTACTTCATTCTGAAAATTGTACAATTCTAATATTTCTAGGTAC3'	Constructing pro-sHsp22.7-pABAi
sHsp22.7-HSE-R	5'CTAGAAATATTAGAATTGTACAATTTTCAGAATGAAGTA3'	
Hsp80-HSE-F	5'AGCTTATTTCTGAAAATCAATAGAAAGAACACAAGGCAGAAGAAAAGGTAC3'	Constructing pro-Hsp80-pABAi
Hsp80-HSE-R	5'CTTTTCTTCTGCCTTGTGTTCTTTCTATTGATTTTCAGAAATA3'	
sHsp17.6-HSE-F	5'AGCTTGAGTGAAGAAGCTTCAAGGAGAGAAAAAAGGTAC3'	Constructing pro-sHsp17.6-pABAi
sHsp17.6-HSE-R	5'CTTTTTTCTCTCCTTGAAGCTTCTTCACTCA3'	
sHsp21LUC-F	5'GGGGTACCATCAACATTCTTTTTTTTTTCTC3'	Constructing pro-sHsp21-pGreenII0800
Hsp21LUC-R	5'ACGCGTCGACTTTATAATTATATTTGATAAATGAAG3'	
Hsp80LUC-F	5'GGGGTACCCCTTAGGTAGAGGGACAGTTTATC3'	Constructing pro-Hsp80-pGreenII0800
sHsp80LUC-R	5'ACGCGTCGACTACAGATCTGCGATGCTGC3'	
sHsp22.7LUC-F	5'GGGGTACCGTAATAAGCTTTCGTAATACATAG3'	Constructing pro-sHsp22.7-pGreenII0800
sHsp22.7LUC-R	5'ACGCGTCGACTTTTTTTTTTTCCTGATTTGATG3'	
sHsp17.6LUC-F	5'GGGGTACCAGATAAGCAAGTTGCAAAGTC3'	Constructing pro-sHsp17.6-pGreenII0800
sHsp17.6LUC-R	5'ACGCGTCGACTTTCAGTGAGCTTTTGAAAAAC3'	
